# RNA-Binding Proteins in Cardiomyopathies

**DOI:** 10.3390/jcdd11030088

**Published:** 2024-03-05

**Authors:** De-Li Shi

**Affiliations:** 1 Department of Medical Research, Affiliated Hospital of Guangdong Medical University, Zhanjiang 524001, China; de-li.shi@upmc.fr; 2 Laboratory of Developmental Biology, Centre National de la Recherche Scientifique, UMR7622, Institut de Biologie Paris-Seine (IBPS), Sorbonne University, 75005 Paris, France

**Keywords:** cardiomyopathy, RNA-binding protein, post-transcriptional regulation, RBM20, RBM24, RBPMS, RBFOX, IGF2BP2, YTHDC1

## Abstract

The post-transcriptional regulation of gene expression plays an important role in heart development and disease. Cardiac-specific alternative splicing, mediated by RNA-binding proteins, orchestrates the isoform switching of proteins that are essential for cardiomyocyte organization and contraction. Dysfunctions of RNA-binding proteins impair heart development and cause the main types of cardiomyopathies, which represent a heterogenous group of abnormalities that severely affect heart structure and function. In particular, mutations of RBM20 and RBFOX2 are associated with dilated cardiomyopathy, hypertrophic cardiomyopathy, or hypoplastic left heart syndrome. Functional analyses in different animal models also suggest possible roles for other RNA-binding proteins in cardiomyopathies because of their involvement in organizing cardiac gene programming. Recent studies have provided significant insights into the causal relationship between RNA-binding proteins and cardiovascular diseases. They also show the potential of correcting pathogenic mutations in RNA-binding proteins to rescue cardiomyopathy or promote cardiac regeneration. Therefore, RNA-binding proteins have emerged as promising targets for therapeutic interventions for cardiovascular dysfunction. The challenge remains to decipher how they coordinately regulate the temporal and spatial expression of target genes to ensure heart function and homeostasis. This review discusses recent advances in understanding the implications of several well-characterized RNA-binding proteins in cardiomyopathies, with the aim of identifying research gaps to promote further investigation in this field.

## 1. Introduction

Cardiomyopathies are structural and functional abnormalities of the myocardium and represent a heterogenous group of cardiac disorders, including dilated cardiomyopathy (DCM), hypertrophic cardiomyopathy (HCM), restrictive cardiomyopathy (RCM), and arrhythmogenic cardiomyopathy (ACM) [1,2,3]. Although these cardiomyopathy phenotypes are characterized by distinct morphological and functional traits [4], they are all closely associated with heart failure, which represents the clinical syndrome of these cardiovascular diseases, as well as the most frequent cause of hospitalization and death worldwide [5,6]. Mutations or the dysregulated expression of cardiac myofibrillar structural and functional proteins are linked to the pathogenesis of cardiomyopathies [3]. In particular, truncated variants of the giant sarcomeric protein TITIN, which has an important structural role and signaling function in cardiac physiology and disease [7], have been identified as the main genetic cause of DCM [8,9,10,11,12]. The switch from the fetal to adult TITIN isoform plays an important role in cardiac stiffness and is mediated by splicing factors that belong to the family of RNA-binding proteins (RBPs). It is well established that RNA-binding motif protein 20 (RBM20) regulates *TITIN* alternative splicing and that its mutations are responsible for the DCM phenotype in humans [13]. Increasing evidence suggests that other RBPs also critically contribute to the alternative splicing of sarcomeric genes. Thus, their dysfunctions are potentially involved in cardiomyopathies [14,15,16].

RBPs are major regulators of gene expression at the post-transcriptional level [17] and contribute to generating protein abundance and diversity within a cell [18,19]. They not only show dynamic expression between fetal and adult hearts under healthy conditions, but also become reactivated or repressed in heart failure [20,21], suggesting their important roles in promoting cardiac differentiation and maintaining cardiac homeostasis. Loss or the dysregulated activity of several RBPs have been closely associated with cardiomyopathies in humans or in animal models, as exemplified by RBM20 mutations in DCM patients [13]. With the expanded identification of the cardiac-specific RBPome, the contribution of RBP-mediated post-transcriptional regulation of cardiac-specific gene expression networks has gained considerable interest for better understanding the complexity of heart development and function [22,23,24,25,26]. Importantly, correcting pathogenic mutations in RBPs or manipulating their activity has the strong potential to rescue cardiomyopathy or promote cardiac regeneration after myocardial injury [27,28,29]. Therefore, RBPs have emerged as promising targets for therapeutic interventions for cardiovascular dysfunction [26,30]. Recent studies have not only further established the causal relationship between RBPs and cardiomyopathies but have also provided novel insights into the mechanism underlying disrupted cardiomyocyte structural and functional gene expression due to RBP dysfunction. Our knowledge on RBP-regulated heart development and cardiovascular disease is rapidly evolving. An update of current advances in this field will help to identify research gaps and promote future investigation.

## 2. RBPs Associated with Cardiomyopathies

The identification of gene mutations in human patients, associated with functional studies using reverse genetic approaches in animal models, such as mouse and zebrafish, has uncovered conserved as well as species-specific functions of RBPs in cardiac development. The following sections will discuss the recent understanding of well-characterized RBPs, including RBM20, RBM24, RBPMS, RBPMS2, RBFOX1, RBFOX2, and several readers of mRNA methylation, such as IGF2BP2 and YTHDC1, associated with cardiomyopathy in humans and/or animal models.

### 2.1. RBM20 Mutations Disrupt Cardiac-Specific Alternative Splicing in Cardiomyopathies

**Figure 1:**
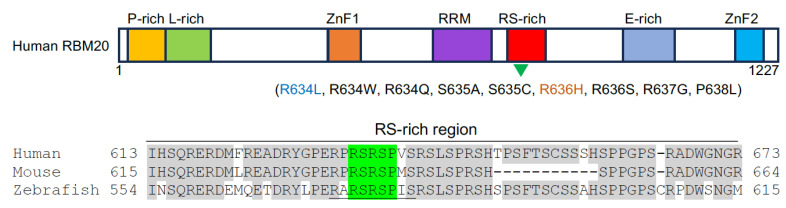
Schematic representation of human RBM20 protein structure. Functional domains are indicated by colored boxes (P-rich, proline-rich region; L-rich, leucine-rich region; E-rich, glutamate-rich region; ZnF, zinc finger domain) and the alignment of amino acids in the RS-rich region from three vertebrate species (human, NP_001127835.2; mouse, NP_001164318.1; zebrafish, XP_021325295.1). Several validated pathogenic mutations in the RSRSP stretch are shown under the schema (green arrowhead). Conserved residues are shadowed in grey. The RS-rich region is indicated in green and the putative nuclear localization signal in the RS-rich region is underlined [41]. Note that the R634L variant (blue) co-segregates with left ventricular non-compaction cardiomyopathy [46] and the R636H variant (orange) may be also associated with HCM [47].

RBM20 contains two putative zinc finger domains and a central RNA recognition motif (RRM), followed by a highly conserved arginine/serine (RS)-rich region (Figure 1). The RRM binds to RNAs with a UCUU sequence to regulate alternative splicing [31]. Since the first report on *RBM20* as a DCM-related gene [32], a large number of heterozygous missense mutations, mainly located at an arginine-serine-arginine-serine-proline (RSRSP) stretch within the RS-rich region, have been identified in DCM patients [33,34,35], and their pathogenic effects have been recently validated in animal models or by using an induced pluripotent stem cell line [28,36,37,38,39,40,41]. These mutations perturb alternative splicing of the *TITIN* gene and many other targets, including the DCM-related *CAMK2D* (calcium/calmodulin-dependent protein kinase type II delta), leading to disrupted cardiac stiffness and abnormal intracellular calcium handling [13,34,35,42]. It has been shown that changes in the RSRSP stretch affect its phosphorylation, which may be important for the nuclear localization of RBM20 and are causative in DCM [43,44]. Indeed, RBM20 proteins with mutated RS-rich regions display sarcoplasmic localization and accumulate in cytoplasmic processing bodies, thus preventing their nuclear function [40,41,45]. Recent works have proposed other mechanisms underlying the mis-localization of RBM20 RS-domain variants. One study suggests that the RS domain represents a nuclear localization signal that mediates RBM20 nuclear transport independently of phosphorylation [41]. Another study shows that disruption in the RS region impairs the physical interaction of RBM20 with Transportin-3, which functions as a nuclear importer of RBM20 [40]. Although multiple mechanisms may account for the dysregulated post-transcriptional activity of mutant RBM20 proteins, it is particularly interesting that correcting these pathogenic variants through precise base editing or promoting the interaction of RBM20 with its nuclear importer can redirect RBM20 nuclear localization and rescue DCM, at least in animal models [27,28,40].

The human *TITIN* gene contains 364 exons with 363 coding ones. It produces various protein isoforms with different functions through alternative splicing [48]. Cardiac muscle expresses two main TITIN isoforms: a more compliant N2BA isoform with a longer extensible I-band region and a stiff N2B isoform, which serve as long and short molecular springs, respectively [49]. Different proportions of these isoforms expressed in fetal and adult hearts confer the passive stiffness of cardiac muscle [48,50]. However, changes in their relative levels can affect cardiac function and lead to DCM. RBM20 functions as a splicing repressor of *TITIN* pre-mRNA [51,52]. It prevents the inclusion of exons 51–218, which encode I-band regions, and leads to the expression of the shorter and stiffer N2B isoform of TITIN [10]. Thus, the dysfunction of RBM20 affects TITIN isoform switching during heart development and disrupts cardiomyocyte stiffness. Nevertheless, mis-localized pathogenic RBM20 variants in DCM not only retain their splicing activity [40], but also mediate distinct mRNA interactions to alter other processes of post-transcriptional regulation that occur in the cytoplasm, such as circular RNA production and alternative polyadenylation [45]. Therefore, RBM20 mutations can cause DCM through splicing-dependent and splicing-independent mechanisms.

There is also evidence that RBM20 may be a candidate gene for HCM [47,53]. Exome sequencing of HCM patients has identified deleterious variants of RBM20, but it seems that these mutations affect structural and functional domains other than those identified in DCM patients [53]. Thus, it is still unclear how they change RBM20 localization and post-transcriptional regulatory activity. A more recent report shows that the R636H pathogenic variant of RBM20 associated with DCM is also present in HCM [47]. These observations suggest that, to some extent, RBM20 may contribute to HCM through a similar mechanism as in DCM. Further studies are necessary to firmly establish the causal relationship between RBM20 mutations and the pathogenesis of HCM.

### 2.2. RBM24 Is Associated with DCM

**Figure 2:**
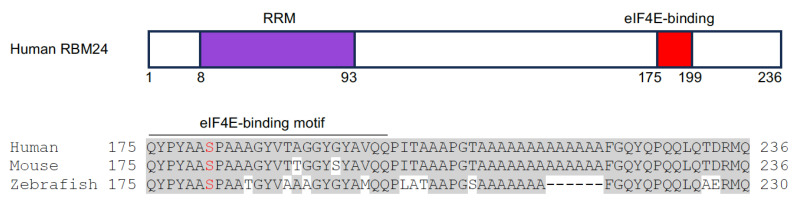
Schematic representation of human RBM24 protein structure and the amino acid alignment of the C-terminal region from three vertebrate species (human, NP_001137414.1; mouse, NP_001074894.1; zebrafish, NP_998030.1). The RRM is indicated in purple and the eukaryotic initiation factor 4E (eIF4E)-binding motif is shown by red color box. Conserved residues are shadowed in grey. The phosphorylable serine residue (S) in the eIF4E-binding motif is indicated in red [61].

Rbm24 is highly conserved from *C. elegans* to mammals [54], harboring a single RRM at the N-terminal region and a more divergent C-terminal half but with several characteristic motifs (Figure 2). The RRM binds to AU/U-rich ligands that are present in a wide spectrum of target mRNAs [55]. In vertebrate embryos, the heart-specific expression of Rbm24 can be detected as early as in the heart fields or the cardiac crescent [56,57,58,59,60], and this is activated after the expression of Nkx2.5 [56], suggesting that Rbm24 may be induced by and function downstream of cardiac-specific transcription factors. Zebrafish have two *rbm24* paralogs, *rbm24a* and *rbm24b*. Rbm24a protein exhibits a higher degree of overall identity and shows an identical expression pattern to Rbm24 in other vertebrates [54,57].

The loss of Rbm24 in zebrafish and mice produces a strong phenotype of heart abnormalities, characterized by defective looping, the occurrence of cardiac edema, and impaired valve development [58,61,62,63]. The knockout of *Rbm24* in mice leads to the complete exclusion or minor inclusion of muscle-specific exons in E11.5 embryos [63]. Importantly, the affected genes are closely associated with cardiogenesis, sarcomere assembly, and the pathogenesis of DCM, and include *Naca*, *Fxr1*, *Abcc9*, *Usp25*, and *Usp28*. This is further supported by the observation that the knockdown of *rbm24a* in zebrafish impairs myofibrillar integrity by disrupting sarcomere organization [60]. Mechanistically, Rbm24 prevents the repression of exon inclusion mediated by PTBP1 (polypyrimidine tract binding protein 1) and hnRNP A1/A2 (heterogeneous nuclear ribonucleoprotein A1/A2) through binding to an intronic splicing enhancer and functioning as an activator of cardiac muscle-specific exon inclusion [63]. The heart-specific conditional knockout of *Rbm24* in postnatal mice disrupts the isoform transition of Titin protein, leading to progressive DCM [64]. Different from RBM20, the loss of RBM24 in human embryonic stem cells or in mice prevents the inclusion of exons coding for the N-terminal Z-repeat domain of TITIN, which binds to the major Z-disc component α-ACTININ 2 [64,65]. In addition, at early stages of induced cardiac differentiation from human embryonic stem cells, RBM24 is also involved in the alternative splicing of *α-ACTININ 2* transcripts. It promotes the inclusion of exon 6, which codes for an actin-binding domain. This facilitates the TITIN-mediated interaction of α-ACTININ 2 with cardiac-specific MYH6, which can subsequently substitute non-muscle MYH10 [65]. Therefore, RBM24 plays a conserved role in cardiac myofibrillogenesis by regulating sarcomere assembly and integrity.

It is of note that Rbm24 is also involved in regulating *CAMK2D* splicing. The deletion of Rbm24 in mice causes aberrant expression of the CAMK2C isoform, leading to alterations in calcium handling and prolongation of the QT interval [66]. Thus, Rbm24 deficiency may cause electrophysiological abnormalities and disrupt cardiac rhythm.

In addition to the regulation of isoform switching for sarcomeric proteins, Rbm24 also functions in other post-transcriptional processes to promote heart development. It has been shown that Rbm24 represents a transcriptional target and a translational regulator of p53 in the mouse embryonic heart. In *Rbm24* homozygous mutant mice, the aberrant activation of p53-dependent apoptosis partly contributes to endocardial cushion defects, growth retardation, and embryonic lethality at E12.5–13.5 [61]. Vertebrate Rbm24 proteins display a highly conserved short amino acid stretch (residues 175–199 in human RBM24), which interacts with eukaryotic initiation factor 4E (eIF4E) to prevent its association with the 5′-cap of *p53* mRNA and assembly of the translation initiation complex, thereby repressing *p53* mRNA translation [61]. However, phosphorylation of the serine residue in the eIF4E-binding motif of Rbm24 releases its interaction with eIF4E, thus rendering it as a translational activator [61]. This suggests that Rbm24 regulates heart development, function, and homeostasis through interaction with its partners and that the eIF4E-binding motif may represent a potential therapeutic target for modulating Rbm24 activity. The regulation of mRNA translation is consistent with the dynamic subcellular localization of Rbm24 during muscle differentiation. At least in skeletal muscle, Rbm24 is localized in the cytoplasm of fate-committed myoblasts but is translocated in the nucleus of differentiated myofibers [67]. Whether and how Rbm24 undergoes cytoplasm-to-nuclear translocation during cardiomyocyte differentiation merits future investigation.

Although the involvement of RBM24 in human heart disease remains to be determined, there is evidence showing the up-regulation of RBM24 in heart failure, which is associated with fetal-specific gene expression and protein isoform switching [20]. Given the strikingly conserved cardiac-specific expression of RBM24 during early development, it will be of interest to decipher the origin and mechanism of cardiac defects due to RBM24 deficiency, such as the impaired differentiation of progenitor cells, the disrupted morphogenesis of heart chambers or endocardial cushions, or the direct dysregulation of cardiac structural and functional genes. There is a possibility that Rbm24 displays dynamic functions dependent on its subcellular localization and its interaction with protein partners.

### 2.3. Loss of RBPMS and RBPMS2 Causes DCM or HCM

RNA Binding Protein with Multiple Splicing (RBPMS) variants and RBPMS2 cluster into two related families of RBPs with an RRM near the N-terminal region [22,68]. The RRM binds tandem CAC motifs with a variable spacer in mRNA targets [69]. RBPMS and RBPMS2 are expressed in the myocardium during development and in the adult of different vertebrate species [68]. Recent studies in vertebrate models have demonstrated their potential involvement in different types of cardiomyopathies. Rbpms plays an important role in post-natal cardiac function. The constitutive knockout of *Rbpms* in mice leads to excessive trabeculation associated with reduced thickness of the ventricles, reminiscent of non-compaction cardiomyopathy [70]. The cardiac-specific deletion of *Rbpms* in mice causes severe defects in cardiomyocyte contraction, resulting in DCM and early lethality in the adult [71]. The loss of Rbpms in the heart also disrupts the alternative splicing of sarcomeric genes, including *Titin* and *Pdlim5*, by preventing the inclusion of exons. In conditional *Rbpms* knockout hearts, the skipping of exons 11 and 47 in the *Titin* transcript leads to the expression of truncated N2BA and N2B isoforms, which may contribute to impaired cardiomyocyte contractility. The same splicing defects are also present in *RBPMS*-deficient cardiomyocytes derived from human induced pluripotent stem cells [71]. This suggests the conserved function of RBPMS in cardiac function and raises the possibility that mutations or the dysfunction of RBPMS may contribute to DCM in humans.

In zebrafish, both *rbpms2a* and *rbpms2b* are enriched in the nkx2.5-positive cell population. The simultaneous loss of *rbpms2a* and *rbpms2b* does not seem to impact cardiac morphogenesis but causes cardiac edema and early cardiac dysfunction with reduced ejection fraction [72]. RNA-seq analysis indicates that Rbpms2 regulates the alternative splicing of several genes involved in cardiac function, such as *mybpc3* and *myom2a*, which encode sarcomeric proteins. Consistently, *rbpms2* mutant zebrafish display disorganized myofibrillar arrays and defective calcium handling [72]. Importantly, MYBPC3 mutations are most frequently detected in patients with HCM [73], and MYOM2 mutations are also identified in patients with HCM, as well as Tetralogy of Fallot [74]. It is of note that *RBPMS2*-deficient human cardiomyocytes also display the same defective myofibril structure and calcium handling as zebrafish *rbpms2* mutants [72], suggesting the conserved molecular and cellular function of this RBP in heart development and disease.

### 2.4. Rbfox1 and Rbfox2 Are Involved in the Pathogenesis of Heart Disease

**Figure 3:**
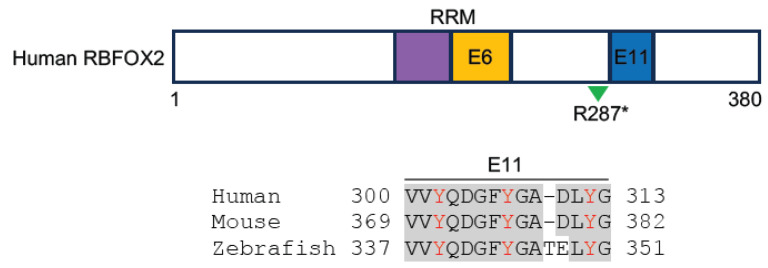
Schematic representation of human RBFOX2 protein structure and the alignment of sequences involved in RBFOX2 subcellular localization and formation of the multiprotein complex (human, NM_001031695.2; mouse, NP_444334.3; zebrafish, XP_021332501.1). These sequences are coded by exon 11 (blue box) in human and mouse and by exon 9 in zebrafish. Conserved residues are shadowed in grey; Tyrosine residues essential for RBFOX2 subcellular localization and complex formation are indicated in red [83,84]. The RRM is indicated in purple- and orange-colored boxes. Splicing-regulated exon 6 (E6), which codes for the second half of the RRM, and the R287* nonsense mutation identified in HLHS patients (green arrowhead) are also shown.

The RNA binding forkhead box homolog (RBFOX) family proteins have three members in vertebrates (Rbfox1, Rbfox2, and Rbfox3). They display an evolutionarily conserved RRM flanked by diversified N- and C-terminal regions (Figure 3). The RRM from all three Rbfox proteins binds to the UGCAUG motif in target mRNAs [75]. Functional studies indicate that Rbfox1 and Rbfox2 are involved in the regulation of heart development and homeostasis. RBFOX1 expression is markedly decreased in human DCM hearts and its deficiency causes pressure overload-induced heart failure in a mouse model. Transcriptomic profiling reveals that Rbfox1 functions as a prominent regulator of cardiac-specific alternative splicing and is required for isoform switching of the transcription factor MEF2 [76]. Consistently, recent studies further show that Rbfox1 mediates mRNA splicing to promote cardiomyocyte maturation [77].

Similarly, RBFOX2 regulates a large program of cardiac-specific alternative splicing events that are important for heart function. In diabetic patients, the disruption of RBFOX2 homeostasis in the heart leads to autoregulation of its exon 6 exclusion [78]. This exon codes for the second half of the RRM, and its in-frame deletion leads to the expression of a truncated protein isoform that acts as a dominant negative mutant to repress Rbfox-regulated alternative splicing [79]. The dysregulation of RBFOX2 activity affects the gene function associated with cytoskeleton and calcium handling, leading to cardiac complications before the onset of diabetic cardiomyopathy [78]. Importantly, the heterozygous loss of *RBFOX2* function results from de novo frameshift, nonsense, or splice site mutations, and the reduced expression of *RBFOX2* due to copy number loss is significantly enriched in patients with hypoplastic left heart syndrome (HLHS) displaying the left ventricle obstruction phenotype [80,81,82,83]. The frameshift mutation of *RBFOX2* is predicted to introduce a premature stop codon, whereas the splice site mutation is expected to include intron 10 in the mRNA. Nonsense mutation truncates a portion of the C-terminal region that includes a sequence coded by an alternative exon 11 (Figure 3). This domain contains several tyrosine residues involved in RBFOX2 subcellular localization [82] and the assembly of a higher-order complex of proteins to enhance RBFOX2 splicing activity [84]. Consistent with the requirement for heart function in humans, the knockout of *Rbfox2* in mice causes DCM, followed by heart failure [85]. Recently, it has been shown that Rbfox2 is involved in regulating the alternative splicing events that influence the communication between cardiomyocytes and the extracellular matrix. The loss of Rbfox2 in mice produces cardiovascular development defects similar to those observed in HLHS [86].

Zebrafish have five *rbfox* genes (*rbfox1*, *rbfox1l*, *rbfox2*, *rbfox3a*, and *rbfox3b*). The knockdown of *rbfox1* alters the cardiac-specific isoform expression of several key proteins regulating cardiomyocyte structure and function [87]. Further confirming the causal relationship of RBFOX2 loss of function with HLHS, zebrafish mutants lacking *rbfox1l* and *rbfox2* develop cardiovascular defects similar to those observed in HLHS patients, including the reduced size and disorganized architecture of ventricular cardiomyocytes, an obstructed aorta, and the absence of endocardial cushions [88]. Mechanistically, the loss of *rbfox1l* and *rbfox2* affects the expression and alternative splicing of genes involved in sarcomere assembly and mitochondrial respiration. It is of note that cardiac defects in *rbfox1l* and *rbfox2* mutants can be rescued by human RBFOX2 but not by HLHS-related RBFOX2 mutant variants [88]. Thus, these observations demonstrate the conserved functions of RBFOX proteins in heart development and cardiovascular disease.

### 2.5. RNA-Binding Proteins Associated with RNA Methylation in Heart Disease

N6-methyladenosine (m6A) is thought to be the most abundant modification in eukaryotic mRNAs, and this modification plays a central role in the post-transcriptional regulation of gene expression [89,90]. It is a dynamic and reversible process coordinated by methyltransferases, such as METTL3/14/16, and demethylases, including FTO and ALKBH5. Several RBP readers, including IGF2BP1/2/3, YTHDC1/2, YTHDF1/2/3, and hnRNPA2B1/C, function to recognize m6A-modified target transcripts and contribute to their alternative splicing, translocation, stability, and translation [90]. Evidence is accumulating that the regulation of mRNA modification is closely associated with cardiovascular disease [91].

IGF2BP2 (insulin-like growth factor 2 mRNA-binding protein 2) shows higher expression in the hearts of DCM patients [92]. The transgenic expression of human IGF2BP2 in mouse hearts causes DCM by inhibiting the expression of sarcomeric and mitochondrial proteins, leading to disorganized sarcomeres and fragmented mitochondria [92]. Nevertheless, the overexpression of IGF2BP2 has no effect on the expression of Titin and Mybpc3, suggesting that it regulates distinct sets of sarcomeric genes. There is a possibility that IGF2BP2-induced cardiac remodeling represents an adaptive response to cardiac stress. Yet, it is still unclear whether this is mediated by interactions with the methylation machinery. Thus, the post-transcriptional mechanism underlying IGF2BP2 function in heart disease requires further investigation, and it will be interesting to examine whether modulating the expression or activity of IGF2BP2 could rescue cardiac dysfunction in DCM.

The cardiac-specific conditional knockout of *YTHDC1* in mice causes DCM by inducing left ventricular chamber enlargement and disorganized sarcomere arrangement, leading to decreased cardiomyocyte contractility and severe systolic dysfunction [93]. Mechanistically, YTHDC1 binds to m6A-modified *Titin* mRNA, and the loss of YTHDC1 increases the expression ratio of N2BA to N2B Titin isoforms [93]. This suggests that YTHDC1 may be involved in regulating cardiac stiffness by facilitating the inclusion of exons that code for the more compliant N2BA isoform. Although it remains to be determined whether other m6A readers of the methylation machinery are involved in cardiomyopathy, several lines of recent evidence indicate that YTHDF1, YTHDF2, and YTHDF3, which either promote mRNA translation or reduce mRNA stability [89], are required for heart development and function. YTHDF1 and YTHDF3 coordinate the differentiation of cardiomyocytes from mouse embryonic stem cells [94], and YTHDF1 can suppress cardiac hypertrophy in an m6A-dependent manner [95]. The loss of YTHDF2 in the cardiomyocytes of adult mice leads to abnormal cardiac remodeling and cardiac function by disrupting the stability and translation of its m6A-modified target transcripts, suggesting that it plays a role in cardiac homeostasis [96,97]. There is also evidence showing that hnRNPC is up-regulated in the sarcomeric Z-disc and likely plays a role in the pathological remodeling of the extracellular matrix [98]. Altogether, these data suggest that the interactions between m6A readers and their mRNA targets contribute to maintain cardiomyocyte integrity and cardiac contractile function.

## 3. Discussion

Accumulating evidence has shed light on the RBP-mediated post-transcriptional regulation of gene expression in heart development and disease. Notably, RBPs contribute to establish an RNA regulatory network that is essential for cardiomyocyte formation and integrity, cardiac contraction, and the maintenance of cardiac homeostasis. Several RBPs, including RBM20 and RBFOX2, are associated with human cardiomyopathies. Functional studies in different vertebrate models have also demonstrated the importance of several well-characterized RBPs, including Rbm24, Rbpms, Rbpms2, Rbfox1, Igf2bp2, and Ythdc1, in regulating the intrinsic post-transcriptional circuit governing sarcomere assembly, mitochondrial respiration, cardiomyocyte organization, and extracellular matrix remodeling (Table 1). Therefore, they are potential candidate genes for cardiovascular diseases. Further studies are necessary to explore how their dysfunctions may be associated with different types of human cardiomyopathies.

It is of note that cardiomyopathy-associated RBPs display both redundant and specific regulatory functions in cardiac alternative splicing, which is important for the expression and switching of cardiac-specific protein isoforms. Therefore, the interplay between different RBPs coordinates the alternative splicing events of common and specific target genes, whereas variation in the expression levels of individual RBPs may cause aberrant splicing and may contribute to cardiac disease [99]. It has been shown that Rbm20 and Rbm24 physically interact to regulate alternative splicing of the *Enigma homolog* (*Enh*) gene, also known as *Pdlim5*. Although Rbm20 or Rbm24 individually display weak or no activity on *Enh* splicing, they cooperate to promote expression of the Enh3 and Enh4 isoforms of Enh, which lack the LIM domain and function to repress cardiac hypertrophy [100]. It is also worth mentioning that many other RBPs, which are not discussed in this review, are involved in regulating the alternative splicing of cardiac genes, although no cardiomyopathy phenotypes were firmly associated with loss of their function. For example, the cardiomyocyte-specific deletion of *Mbnl1* and *Mbnl2* in mice causes heart spliceopathy [101], and the conditional knockout of *QKI* in the cardiomyocytes of adult mice leads to heart failure by disrupting the alternative splicing of cardiac genes with functions in the sarcomere and cytoskeleton [102]. Thus, we may expect that their combined actions coordinate alternative splicing events in heart development, function, and homeostasis. Indeed, it is suggested that, similar to the loss of Rbm24, aberrant splicing due to the dysregulated expression of MBNL and QKI could contribute to the disease mechanism underlying the pathogenesis of heart failure [99]. Since many RBPs are dysregulated in cardiac hypertrophy or heart failure, modulating their expression or activity may represent a potential to rescue cardiovascular dysfunction [103].

Although RBPs act as prominent regulators of cardiac-specific alternative splicing, other mechanisms of post-transcriptional regulation may be also defective following the loss of their function. In this regard, it has been shown that loss of Rbm24 leads to cardiac malformations by promoting *p53* mRNA translation and the aberrant activation of p53-dependent apoptosis [61]. Indeed, Rbm24 can regulate the expression of target transcripts in murine and rat cardiomyocytes through multiple mechanisms, including alternative 3-UTR length, alternative start site, and mRNA destabilization [104]. Similarly, in human embryonic stem cells and cardioids, Rbpms controls a specialized mRNA translation circuit to specify cardiac mesoderm and promote cardiac morphogenesis [105]. In H9c2 rat myoblasts, the deletion of *Rbfox2* affects the expression of contractile and mitochondrial genes via alternative polyadenylation [106]. Igf2bp2 generally functions as an m6A reader and regulates mRNA stability [89]. Although the post-transcriptional mechanism by which its up-regulation induces cardiac remodeling remains unclear, there is a possibility that this induces DCM by disrupting the translation of target mRNAs [92]. Thus, RBPs function in a manner that is dependent on developmental stages, subcellular localization, and physiological or pathological conditions. Further studies are necessary to identify the protein partners and mRNA targets of RBPs for better understanding of the post-transcriptional mechanisms underlying their context-dependent regulatory activity in heart development and cardiomyopathy.

It is worth mentioning that the identification of gene mutations in human patients, combined with mechanistic analyses in animal models, has greatly contributed to our understanding of the RBP-regulated post-transcriptional mechanisms in heart disease. In addition to different mammalian models, zebrafish have become particularly attractive for the live imaging of heart morphogenesis and cardiac dysfunction. Though with a two-chambered heart, zebrafish display largely conserved core regulatory circuits and the cellular lineages essential for cardiac development [107,108]. Therefore, they are widely used for modeling human cardiac disease, with advantages and limitations [109,110]. By employing appropriate and effective experimental strategies, zebrafish represent one of the ideal models for investigating the origin of cardiac defects [111].

## 4. Conclusions

RBPs are clearly involved in the pathogenesis of cardiomyopathies, but the challenge remains to decipher the molecular mechanisms underlying their dysfunctions associated with dysregulated target gene expression. Recent studies have not only identified new candidate RBPs for cardiomyopathies but have also provided further mechanistic insights into the causal relationship between mutations or dysfunctions in RBPs and cardiac disease. Importantly, the possibility of correcting pathogenic mutations in RBPs or manipulating their activity presents a strong potential to rescue cardiomyopathies or promote cardiac regeneration. Therefore, RBPs have emerged as promising targets for therapeutic interventions for cardiovascular dysfunction.

## Figures and Tables

**Table 1:** Brief summary of the RBPs associated with cardiomyopathies in humans and/or animal models.

RBPs	Mutations or Knockout	Cardiovascular Phenotypes	Dysregulated Target Genes	References
RBM20	Heterozygous missense mutations in human patients	DCM and likely HCM in humans	Defective splicing of *Titin*, *Camk2d*, and many other sarcomeric genes	[13,32,42,47,53]
RBM24	Knockout in mice, zebrafish, and human embryonic stem cells	DCM and prolongation of the QT interval in mice	Defective splicing of *Titin*, *Camk2d*, *α-actinin 2*, and other muscle-specific genes; increased *p53* mRNA translation	[61,64,65,66]
RBPMS	Knockout in mice and human cardiomyocytes	Non-compaction cardiomyopathy and DCM in mice	Defective splicing of *Titin*, *Pdlim5*, and cardiac myofibrillogenesis genes	[70,71]
RBPMS2	Knockout in zebrafish and human cardiomyocytes	Cardiac defects in zebrafish reminiscent of HCM	Defective splicing of *Rbfox2, Mybpc3*, *Slc8a1*, and *Myom2a*	[72]
RBFOX1	Knockout in mice and knockdown in zebrafish	Cardiac hypertrophy, cardiomyoapthy, and heart failure	Defective splicing of *Mef2* in mice, and *huG*, *actn3a*, *ptpla*, *camk2g1*, and *ktn1* in zebrafish	[76,87]
RBFOX2	De novo frameshift, nonsense, or splice site mutations in HLHS human patients; knockout in mice and zebrafish	HLHS in humans and zebrafish (*rbfox1/2* mutants); DCM in mice	Defective splicing of sarcomere components (*tpm1*, *tpm3*, and *tnnt3b*), MICOS complex components (*mic19a* and *mic19b*), and cytoskeletal components (*pdlim5b*, *alcama*, and *fmnl3*)	[80,81,82,83,85,88]
IGF2BP2	Overexpression in mice	DCM in mice	Reduced expression of sarcomeric and mitochondrial proteins (Titin and Mybpc3 unaffected)	[92]
YTHDC1	Knockout in mice	DCM in mice	Defective splicing of *Titin*	[93]

## Data Availability

No new data were created in this work.
